# Regulation of Romantic Love Feelings: Preconceptions, Strategies, and Feasibility

**DOI:** 10.1371/journal.pone.0161087

**Published:** 2016-08-16

**Authors:** Sandra J. E. Langeslag, Jan W. van Strien

**Affiliations:** 1 Department of Psychological Sciences, University of Missouri–St. Louis, St. Louis, Missouri, United States of America; 2 Department of Psychology, University of Maryland, College Park, Maryland, United States of America; 3 Institute of Psychology, Erasmus University Rotterdam, Rotterdam, The Netherlands; Vanderbilt University, UNITED STATES

## Abstract

Love feelings can be more intense than desired (e.g., after a break-up) or less intense than desired (e.g., in long-term relationships). If only we could control our love feelings! We present the concept of explicit love regulation, which we define as the use of behavioral and cognitive strategies to change the intensity of current feelings of romantic love. We present the first two studies on preconceptions about, strategies for, and the feasibility of love regulation. Questionnaire responses showed that people perceive love feelings as somewhat uncontrollable. Still, in four open questions people reported to use strategies such as cognitive reappraisal, distraction, avoidance, and undertaking (new) activities to cope with break-ups, to maintain long-term relationships, and to regulate love feelings. Instructed up-regulation of love using reappraisal increased subjective feelings of attachment, while love down-regulation decreased subjective feelings of infatuation and attachment. We used the late positive potential (LPP) amplitude as an objective index of regulation success. Instructed love up-regulation enhanced the LPP between 300–400 ms in participants who were involved in a relationship and in participants who had recently experienced a romantic break-up, while love down-regulation reduced the LPP between 700–3000 ms in participants who were involved in a relationship. These findings corroborate the self-reported feasibility of love regulation, although they are complicated by the finding that love up-regulation also reduced the LPP between 700–3000 ms in participants who were involved in a relationship. To conclude, although people have the preconception that love feelings are uncontrollable, we show for the first time that intentional regulation of love feelings using reappraisal, and perhaps other strategies, is feasible. Love regulation will benefit individuals and society because it could enhance positive effects and reduce negative effects of romantic love.

## Introduction

Romantic love strikes virtually everyone at least once (i.e., its lifetime prevalence approaches 100%) [1] and has a great impact on our lives. Romantic love has positive effects on individuals and society as a whole. For example, love is associated with positive emotions such as euphoria [2] and romantic relationships enhance happiness and life satisfaction [3]. But love also has a negative impact on individuals and society. For example, love is associated with stress [4] and jealousy [5], and romantic break-ups are associated with sadness and shame [6], a decrease in happiness and life satisfaction [7], and depression [8]. The high prevalence of love combined with its significant positive and negative impact on individuals and society make it an important research topic.

The word ‘love’ has many different meanings and may have different meanings to different people. Researchers have proposed several taxonomies of love, with various numbers of love types or components [9–13]. In this study, two types of love feelings are considered: infatuation and attachment. Infatuation is the overwhelming, amorous feeling for one individual, and is similar to the concepts ‘passion’ or ‘infatuated love’ [10], ‘romantic love’ [11], ‘passionate love’ [12], and ‘attraction’ [13]. Attachment, on the other hand, is the comforting feeling of emotional bonding with another individual, and is similar to the concepts ‘intimacy’ with ‘decision/commitment’ [10], and ‘companionate love’ [10–12].

Love feelings are sometimes weaker than desired. Infatuation is typically most intense at the early stages of love after which it decreases relatively quickly [13–15] and attachment takes some time to develop [13–15] after which it decreases over the course of decades [16]. The decrease of infatuation and attachment over time threatens the stability of romantic relationships. Indeed, falling out of love is the primary reason for divorce [17]. Love feelings can also be stronger than desired. People may, for example, be in love with someone who does not love them back or who has broken up with them. Clearly, it would be advantageous if we could regulate love feelings at will, so that we could up-regulate them when they are weaker than desired, and down-regulate them when they are stronger than desired.

We define love regulation as the use of behavioral or cognitive strategies to change the intensity of current feelings of romantic love. In an interview study, participants reported that their love feelings were involuntary and uncontrollable [2]. Nevertheless, three lines of research suggest that love regulation may actually be feasible. First, it is well known that people can regulate their emotions [18–21], which entails generating new emotions or changing the intensity of current emotions using behavioral or cognitive strategies [20]. There are multiple emotion regulation strategies, including situation selection, distraction, expression suppression, and cognitive reappraisal. Situation selection is to avoid or seek out certain situations to change the way you feel (e.g., attending a party to have fun) [21]. Distraction entails performing a secondary task to reduce the intensity of emotions (e.g., playing a video game to forget about a bad incident at work) [20]. Expression suppression involves inhibiting the expression of an emotion (e.g., keeping a poker face) [22]. Cognitive reappraisal involves reinterpreting the situation to change the way you feel (e.g., decreasing or increasing nervousness by reinterpreting an upcoming job interview as an opportunity to learn more about the company or as a once in a lifetime opportunity, respectively) [22]. Emotion regulation can be used to up- and down-regulate positive and negative emotions [23] and may happen implicitly or explicitly [18].

However, love is sometimes considered a motivation (or drive) rather than an emotion [24]. One reason why love would not be an emotion is that it elicits different emotions depending on the situation. Reciprocated love, for example, may elicit the emotion euphoria, while unreciprocated love may elicit the emotion sadness. It is therefore important that a second line of research has shown that people can use cognitive strategies to regulate their motivations, including sexual arousal [25], excitement about monetary reward [26–29], and craving for alcohol, food, and cigarettes [30, 31]. The evidence that motivations can be regulated intentionally supports the idea that explicit love regulation may be feasible.

Finally, a third research line has shown that people think more favorably of their romantic partner than objectively justified [32, 33]. Importantly, people who idealize their partner and whose partners idealize them have happier relationships [34]. These findings suggest that implicit up-regulation of love feelings for the current romantic partner is feasible and that it contributes to relationship satisfaction.

Even though this last research line shows that people can regulate their love feelings implicitly, there are no studies that provide information about the deliberate, explicit up- and down-regulation of love feelings. In two studies, we systematically examined preconceptions about, strategies for, and the feasibility of explicit regulation of love feelings. The first goal was to determine whether people think that love feelings can be controlled or not. Participants answered a series of questions that measured perceived control over love feelings and previous research [2] led us to hypothesize that people would perceive love feelings as uncontrollable. The second goal was to reveal which strategies people use when they try to up- and down-regulate their love feelings. Participants responded to four open questions and we expected that people would report the use of typical behavioral and cognitive emotion regulation strategies mentioned above. First, we conducted a pilot study (Study 1) and then we conducted another study (Study 2) to confirm the findings of the pilot study.

In addition, Study 2 employed a love regulation task to achieve the final research goal, which was to examine if people can intentionally up- and down-regulate love feelings. In this first empirical test of the feasibility of love regulation, we focused on the reappraisal strategy because it is considered effective in altering feeling intensity and beneficial for cognitive and social functioning [21]. Situation-focused reappraisal entails changing the emotional meaning of a situation by reinterpreting it [21], for example by focusing on positive or negative aspects of the situation, or by imagining a positive or negative outcome [35]. The use of cognitive reappraisal to regulate love feelings is related to the notion that cognitive processes, including making attributions, are associated with relationship satisfaction [36, 37]. We focus on the intensity of infatuation and attachment rather than relationship outcomes, since love feelings do not occur exclusively in the context of romantic relationships [14].

Because it depends on the situation whether people would benefit from love up- or down-regulation, we tested a group of people who were involved in a romantic relationship and a group of people who had recently experienced a romantic break-up. People who are currently in a romantic relationship were expected to benefit from love up-regulation, because that would stabilize their relationship. People who have just experienced a break-up, in contrast, would benefit from love down-regulation, because that could help them cope with the break-up. Because previous research has shown that intense feelings of romantic love can be elicited by viewing pictures of the beloved [38], pictures of the (ex-)partner were used to elicit feelings of love, which participants were instructed to regulate in an explicit regulation task. Because self-reports are the only way to assess phenomenology (i.e., how someone feels) [39], participants rated how infatuated and how attached they felt after each regulation condition. It was hypothesized that love up-regulation would increase feelings of infatuation and attachment, whereas love down-regulation would decrease feelings of infatuation and attachment in both groups. It is of course important to dissociate between the concept of love regulation and the well-established concept of emotion regulation. Therefore, participants also rated how negative or positive they felt after each regulation condition. It was expected that love up-regulation would make the relationship group feel more positive, while love down-regulation would make them feel more negative. The opposite pattern was expected for the break-up group: feeling more negative following up-regulation and more positive following down-regulation. This hypothesis shows how love regulation is theoretically distinct from emotion regulation. That is, love regulation targets the intensity of love feelings rather than emotions. Of course, the change in love feelings may in turn influence emotions or affect. The direction of the effect of love regulation on emotion or affect may differ depending on the context, as indicated by the hypothesized opposite effects of love regulation on emotion/affect in the relationship and break-up groups.

Even though self-reports gain a unique insight into what people experience, they also suffer from social desirability biases and demand characteristics [40, 41]. Therefore, in addition to subjective self-reports, we used event-related potentials (ERPs) as a more objective measure of love regulation success. ERPs have been used before to study emotion regulation and to study romantic love, but not to study love regulation. The late positive potential (LPP) reflects multiple and overlapping positivies over the posterior scalp beginning in the time range of the classic P300, i.e., around 300 ms after stimulus onset. The LPP amplitude is typically enhanced for negative and positive compared to neutral stimuli [19] and is therefore thought to reflect the affective and motivational intensity of information and the resulting motivated attention [42]. Correspondingly, we have shown that the LPP is enhanced in response to pictorial and verbal beloved-related information compared to control information [32, 43, 44]. Importantly, the LPP amplitude is modulated by emotion regulation instructions according to the regulatory goal: emotion down-regulation reduces the LPP amplitude, while emotion up-regulation enhances the LPP amplitude [27, 45–49]. The LPP amplitude can therefore be used as an objective measure of regulation success [19]. Because the LPP reflects affective and motivational significance and the resulting motivated attention, rather than valence [42], regulation effects in the LPP amplitude reflect how regulation changes the affective and motivational intensity of information and the amount of motivated attention allocated to that information. It was expected that love up-regulation would enhance the LPP in response to pictures of the (ex-)partner in both groups, which would indicate that love up-regulation would enhance the affective and motivational significance of, and the resulting motivated attention to the (ex-)partner. Love down-regulation, in contrast, was expected to reduce the LPP amplitude to (ex-)partner pictures in both groups, which would indicate that love down-regulation would reduce the affective and motivational significance of, and the resulting motivated attention to the (ex-)partner.

## Study 1 –Methods

### Participants

Thirty-two participants (18–30 yrs, *M* = 21.4, 7 men) who were in love by self-report were recruited from the University of Maryland community in the US. Being in love was an inclusion criterion because some of the questions assessing perceived control over love feelings (see below) contained a blank in which the participants had to mentally insert the name of their beloved. The study was approved by the Institutional Review Board of the University of Maryland and written informed consent was obtained. Participants were remunerated with $10.

### Procedure

First, participants completed some questions about their love feelings and their romantic relationship [44]. Participants also completed the Infatuation and Attachment Scales (IAS) [14], and the Passionate Love Scale (PLS) [50] to assess the intensity of infatuation and attachment. Then, participants completed 17 questions to assess perceived control of love feelings (Cronbach’s alpha = .93), see the S1 Appendix. These questions were phrased to measure perceived control over love in general, and over infatuation and attachment specifically. They were also phrased to measure perceived control over one’s own vs. people’s love feelings, and over the intensity and object of love feelings. Participants responded using a 9-point Likert scale (1 = totally disagree, 9 = totally agree).

Subsequently, participants answered four open questions about the use of behavioral and cognitive strategies in the contexts of heartbreak and long-term relationships. We distinguished between emotion regulation and love down-regulation in the context of heartbreak by asking two questions: “What do you do or think to feel better when you have a broken heart?” (i.e., emotion regulation), “What do you do or think to decrease feelings of love when you have a broken heart?” (i.e., love down-regulation). In addition, we distinguished between maintaining relationships and love up-regulation in the context of long-term relationships by asking two questions: “What do you do or think to maintain a long-term relationship?” (i.e., maintaining relationships), and “What do you do or think to prevent that feelings of love decline in a long-term relationship?” (i.e., love up-regulation). If participants had not experienced heartbreak or any long-term relationships, they replied with what they think they would do in those circumstances.

### Analyses

The mean score on the 17 perceived control questions was subjected to a one-sample *t*-test against 5, to test if it differed from neutral. In addition, responses on subsets of the 17 questions that measured perceived control over a certain aspect of love were averaged to obtain measures of perceived control over seven different aspects of love (love in general, infatuation, attachment, self, people in general, intensity of love, and object of love). Five paired sample *t*-tests were conducted to test for differences in perceived control between related aspects of love (i.e., love in general vs. infatuation, love in general vs. attachment, infatuation vs. attachment, self vs. people in general, and intensity vs. object of feelings).

The responses to the four open strategy questions were analyzed qualitatively. Many participants listed multiple strategies in response to each of the four open strategy questions. Each strategy was scored as being an exemplar of a certain category. A priori categories were emotion regulation strategies such as reappraisal, distraction, and suppression [20, 21]. In the heartbreak context, reappraisal was subdivided into “focus on the negative aspects of the beloved/relationship”, “think of negative future scenarios”, “think about the positive aspects of the situation”, and “other”. In the long-term relationship context, reappraisal was subdivided into “focus on the positive aspects of the beloved/relationship”, and “think of positive future scenarios”. Other categories such as avoidance (see Tables 1 and 2) were added on the basis of participants’ responses.

**Table 1:** Counts and percentages of participants reporting the use of certain regulation strategies in the context of heartbreak.

	Study 1 (*n* = 32)		Study 2			
			Relationship group (*n* = 20)		Break-up group (*n* = 20)	
Strategy	Feel better when broken-hearted	Decrease love feelings	Feel better when broken-hearted	Decrease love feelings	Feel better when broken-hearted	Decrease love feelings
Reappraisal: Focus on negative aspects of beloved/relationship	2 (6%)	10 (31%)	0	9 (45%)	3 (15%)	9 (45%)
Reappraisal: Think of negative future scenarios	0	2 (6%)	0	0	0	0
Reappraisal: Think of positive aspects of the situation	3 (9%)	5 (16%)	1 (5%)	2 (10%)	2 (10%)	1 (5%)
Reappraisal: Other	7 (22%)	12 (38%)	3 (15%)	0	1 (5%)	6 (30%)
Distraction	19 (59%)	6 (19%)	15 (75%)	8 (40%)	16 (80%)	7 (35%)
Avoidance	1 (3%)	6 (19%)	2 (10%)	3 (15%)	2 (10%)	3 (15%)
Expression suppression	0	0	0	0	0	1 (5%)
Social support	17 (53%)	3 (9%)	4 (20%)	0	5 (5%)	1 (5%)
Eating/smoking	3 (9%)	0	3 (15%)	1 (5%)	1 (5%)	0
Express emotions	2 (6%)	1 (3%)	1 (5%)	1 (5%)	0	0
No decrease	0	2 (6%)	0	0	0	2 (10%)
Other	3 (9%)	1 (3%)	1 (5%)	1 (5%)	1 (5%)	0

**Table 2:** Counts and percentages of participants reporting the use of certain regulation strategies in the context of long-term relationships.

	Study 1 (*n* = 32)		Study 2			
			Relationship group (*n* = 20)		Break-up group (*n* = 20)	
Strategy	Maintain long-term relationship	Prevent love decline	Maintain long-term relationship	Prevent love decline	Maintain long-term relationship	Prevent love decline
Reappraisal: Focus on positive aspects of beloved/relationship	2 (6%)	3 (9%)	0	1 (5%)	0	4 (20%)
Reappraisal: Think of positive future scenarios	3 (9%)	1 (3%)	0	0	0	1 (5%)
Communication/honesty	13 (41%)	4 (13%)	9 (45%)	2 (10%)	10 (50%)	4 (20%)
Trust	6 (19%)	1 (3%)	1 (5%)	0	1 (5%)	1 (5%)
Undertake (new) activities	6 (19%)	15 (47%)	11 (55%)	10 (50%)	2 (10%)	13 (65%)
Express love	6 (19%)	5 (16%)	3 (15%)	3 (15%)	3 (15%)	1 (5%)
Spend (quality) time together	4 (13%)	4 (13%)	3 (15%)	3 (15%)	5 (25%)	3 (15%)
Spend time apart	1 (3%)	3 (9%)	2 (10%)	2 (10%)	3 (15%)	1 (5%)
Loving unconditionally/ making compromises	4 (13%)	1 (3%)	4 (20%)	0	8 (40%)	2 (10%)
No decline	2 (6%)	4 (13%)	0	1 (5%)	0	1 (5%)
Other	6 (19%)	5 (16%)	5 (25%)	3 (15%)	4 (20%)	2 (10%)

## Study 1 –Results

### Participant characteristics

All participants had an opposite-sex beloved. Twenty-seven (84%) of the participants reported to be in a relationship with their beloved, which supports the idea that love does not occur exclusively in the context of relationships [14]. See Table 3 for the other love characteristics.

**Table 3:** Participant characteristics. Means (ranges in parentheses).

	Study 1 (*n* = 32)	Study 2			
		Relationship group (*n* = 20)	Break-up group (*n* = 20)	*t(38)*	*p*
**Duration known beloved/partner [months]**	15.1 (2.0–65.0)	36.7 (3.8–78.0)	33.9 (7.5–76.0)	0.4	.69
**Time since start love feelings [months]**	8.9 (1.0–63.0)	29.2 (3.8–68.3)	26.4 (7.0–53.0)	0.5	.63
**Relationship duration [months]**	6.5 (3.0–17.0)	26.7 (0.5–68.3)	21.4 (5.0–47.5)	0.9	.35
**Relationship quality [1–9]**	7.9 (6–9)	7.9 (5–9)	7.3 (6–9)	1.8	.073
**IAS infatuation score [1–9]**	3.4 (1.5–5.6)	2.8 (1.8–5.1)	3.3 (1.6–5.4)	-1.8	.083
**IAS attachment score [1–9]**	5.8 (3.5–6.9)	6.0 (4.2–6.9)	3.7 (1.5–5.7)	7.2	**< .001**
**PLS score [1–9]**	7.2 (4.2–8.4)	6.8 (5.4–8.9)	6.1 (1.2–8.2)	1.7	.10
**Positive affect, past 2 weeks [1–5]**	-	3.8 (2.7–4.8)	3.4 (1.8–5.0)	1.8	.082
**Negative affect, past 2 weeks [1–5]**	-	1.9 (1.0–3.8)	2.5 (1.2–3.7)	-2.5	**.016**
**Positive affect, at start of testing session [1–5]**	-	3.0 (2.0–4.1)	3.2 (1.8–4.5)	-1.2	.24
**Negative affect, at start of testing session [1–5]**	-	1.2 (1.0–1.6)	1.8 (1.1–3.3)	-4.2	**< .001**
**ERQ reappraisal score [1–7]**	-	5.1 (2.8–6.7)	5.1 (3.2–6.7)	-0.2	.88
**ERQ suppression score [1–7]**	-	3.0 (1.0–5.5)	3.3 (1.5–6.0)	-0.6	.58

Note.— = data not collected.

### Perceived control

The mean score on the 17 perceived control questions was 4.5 (*SD* = 1.4). A one-sample *t*-test revealed that this tended to be lower than 5 (= neutral), *t*(31) = -1.9, *p* = .066, which implies that participants perceive love feelings as neither controllable, nor uncontrollable, or as somewhat uncontrollable, if anything. See Table 4 for the mean perceived control over the seven different aspects of love. Participants felt more in control of feelings of attachment than infatuation, *t*(31) = 2.4, *p* = .022. There was no difference between the perceived control over the own vs. people’s feelings, *t*(31) = 0.5, *p* = .64. Participants felt more control over the intensity than the object of their love feelings, *t*(31) = 2.1, *p* = .047.

**Table 4:** Mean (SD) perceived control over seven aspects of love.

	Study 1 (*n* = 32)	Study 2	
		Relationship group (*n* = 20)	Break-up group (*n* = 20)
**Love in general**	4.4 (1.6)	4.2 (1.8)	4.1 (1.9)
**Infatuation**	4.4 (1.4)	4.1 (1.5)	4.2 (1.7)
**Attachment**	4.9 (1.7)	5.7 (1.8)	5.2 (1.3)
**Self**	4.6 (1.7)	4.5 (1.5)	4.2 (1.7)
**People in general**	4.5 (1.3)	4.6 (1.4)	4.7 (1.3)
**Intensity of feelings**	4.8 (1.8)	5.2 (1.7)	4.7 (1.5)
**Object of feelings**	4.3 (1.6)	4.1 (1.6)	4.1 (1.8)

*Note*. 1 = totally uncontrollable, 5 = neutral, 9 = totally controllable

### Strategies

See Tables 1 and 2 for the regulation strategies reported. In the context of heartbreak, participants mostly used distraction, social support, and reappraisal. Distraction (e.g., watching TV, listening to music, focusing on work or school, or exercise) and seeking social support (e.g., talking to or spending time with family/friends) were used more to feel better than to decrease love feelings. In contrast, reappraisal was used more to decrease love feelings than to feel better. Reappraisal by focusing on negative aspects of the beloved was the most popular reappraisal strategy. Examples of other reappraisal strategies were thinking that time will heal, finding someone else to love, or focusing on positive aspects of oneself or one’s life. Thinking about positive aspects of the situation (e.g., focusing on the advantages of being single or being hopeful for the future), as well as avoidance (i.e., not talking about the beloved, getting rid of all pictures, and eliminating all contact) were moderately popular strategies to decrease love feelings. Strategies such as reappraisal by thinking about negative future scenarios (“it just wasn’t meant to last”), eating/smoking, and expressing emotions (“cry”) were used least often. None of the participants reported the use of suppression. Two participants reported that they did not, or could not, decrease love feelings.

In the context of long-term relationships, participants stressed the importance of communication/honesty and undertaking (new) activities with their beloved. Communication/honesty was deemed important for maintaining long-term relationships, whereas undertaking (new) activities with the beloved, which is a situation selection strategy, was mostly used to prevent love feelings from declining. Other strategies such as expressing love feelings to the beloved, trust, spending (quality) time with the beloved, loving unconditionally/making compromises, the two reappraisal strategies, and spending time apart from the beloved were mentioned as well. Six participants stated that love feelings would not decline if the relationship was good and/or that they would end the relationship if love feelings would decline.

In short, several behavioral and cognitive strategies were used in the contexts of heartbreak and long-term relationships. Some of these strategies were the typical cognitive and behavioral emotion regulation strategies, such as reappraisal, distraction, and situation selection. While some strategies seemed specific for feeling better during heartbreak or for maintaining long-term relationships, strategies such as reappraisal by focusing on negative aspects of the beloved or the relationship and undertaking (new) activities with the beloved seemed specific for down- and up-regulation of love feelings, respectively.

## Interim Discussion

The results of this first exploratory study show that people perceive love feelings as neither controllable, nor uncontrollable (or as somewhat uncontrollable, if anything). People did perceive more control over some aspects of love than others and the majority of people reported to use a variety of strategies when heartbroken or when in a long-term relationship. Some strategies seemed specific for changing the intensity of love feelings, rather than for regulating emotions or maintaining relationships. Because this was only a pilot study with mostly female participants, we conducted a follow-up study (Study 2) to replicate and confirm these preliminary findings in a more gender-balanced sample. As mentioned in the introduction, Study 2 also included a love regulation task to test the feasibility of love regulation.

## Study 2 –Methods

### Participants

Twenty participants who were in a romantic relationship (19–25 yrs, *M* = 21.7, 10 men) and 20 participants who had recently experienced a romantic break-up (19–26 yrs, *M* = 21.9, 10 men) were recruited from the Erasmus University Rotterdam community in The Netherlands. For brevity, we will use the words ‘partner’ and ‘relationship’ in the remainder of the paper regardless of whether the relationship was ongoing or had dissolved. Inclusion criteria were normal or corrected to-normal vision, right-handedness (as determined by a hand preference questionnaire [51]), no use of medication known to affect the central nervous system, and no mental disorders. The reason for excluding participants with mental disorders was that many mental disorders are associated with emotion dysregulation [23], which indicates that love regulation may also be different in patients than in healthy controls. Four participants had to be excluded from the EEG analyses because of experimenter error during the EEG recording (*n* = 3) or too many artifacts (*n* = 1, more information below). Therefore the EEG analyses are based on 18 participants who were in a romantic relationship (19–25 yrs, *M* = 21.8, 9 men) and 18 participants who had recently experienced a romantic break-up (19–26 yrs, *M* = 21.7, 8 men). The study was approved by the Psychologie Ethische Commissie of the Erasmus Universiteit Rottterdam and written informed consent was obtained. Participants were remunerated with course credit or €15.

### Questionnaires

In addition to the questions about their love feelings and their romantic relationship [44], the 17 perceived control questions (Cronbach’s alpha = .92), the four open regulation strategy questions, the Infatuation and Attachment Scales (IAS) [14], and the Passionate Love Scale (PLS) [50] used in Study 1, participants completed the Emotion Regulation Questionnaire (ERQ) [22] to assess individual differences in the habitual use of reappraisal and suppression. Participants also completed the Positive Affect Negative Affect Schedules (PANAS) twice, once about the last two weeks and once about this moment [52].

### Stimuli

Participants provided 30 digital pictures of their partner. There were no other requirements than that the pictures had to contain the partner. Therefore, the pictures could display parts of the partner (e.g., just the face) or the whole body of the partner, people other than the partner, a variety of facial expressions, objects, and scenery. The pictures were presented to elicit love feelings [38] and to help the participant come up with negative or positive aspects of the partner/relationship and future scenarios (see below). It is important to note that the variety of information on the pictures does not confound the effects of regulation, because the same 30 partner pictures were presented in each regulation condition. For the same reason, differences in picture content between the two groups could not confound the differences in regulation effects between groups. In addition, the pictures ensure high ecological validity, as the partner is typically encountered in a wide variety of contexts and with varying facial expressions. The neutral stimuli were 30 neutral pictures displaying humans from the International Affective Picture System (IAPS) [53] with neutral normative valence (*M* = 5.4, *SD* = 0.5) and low normative arousal (*M* = 3.5, *SD* = 0.5) ratings, see S1 Text.

### Love regulation task

**Fig 1:**
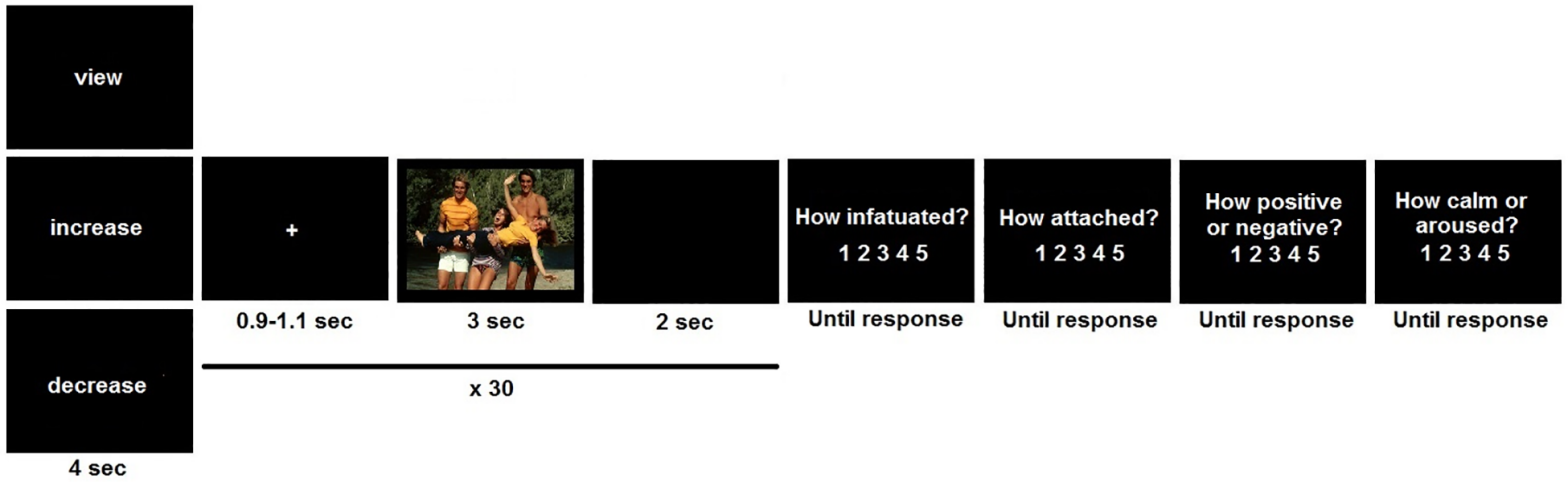
Overview of blocks and trials in the regulation task. Please note that the stimulus in this figure is not actually one of the pictures that were submitted by the participants. Instead, it is an IAPS picture [53] that resembles the kinds of pictures that participants submitted.

Participants completed a love regulation task, see Fig 1, while their electroencephalogram (EEG) was recorded. In the first two blocks, participants passively viewed partner and neutral pictures (order counterbalanced between participants). In the third and fourth block, participants were instructed to up- and down-regulate their love feelings in response to the partner pictures (order counterbalanced between participants) using reappraisal. Up-regulation instructions were to increase love feelings by thinking about positive aspects of the partner (e.g., “He is so funny”) or relationship (e.g., “We get along so well”), or positive future scenarios (e.g., “We’ll get married”). Down-regulation instructions were to decrease love feelings by thinking about negative aspects of the partner (e.g., “She is so lazy”) or relationship (e.g., “We often fight”), or negative future scenarios (e.g., “We won’t stay together forever”). Participants could use the information in the picture for inspiration. For example, the partner wearing a yellow shirt and standing next to a friend on a picture could inspire the participant to up-regulate love feelings by thinking “I love that yellow shirt he’s wearing” and to down-regulate love feelings by thinking “He is always hitting on that friend, and he might cheat on me one day”. These are instructions of situation-focused reappraisal, which involves reinterpreting “the nature of the events themselves, reevaluating others’ actions, dispositions, and outcomes” rather than self-focused reappraisal, which involves altering “the personal relevance of events” ([35], p. 484). That is, focusing on negative/positive aspects of the partner involves a reevaluation of the partner’s dispositions (“My partner is a wonderful person” when focusing on positive aspects vs. “My partner is a terrible person” when focusing on negative aspects), focusing on negative/positive aspects of the relationship involves a reevaluation of the relationship (“I am/was in a good relationship” when focusing on positive aspects, and “I am/was in a bad relationship” when focusing on negative aspects), and imagining positive/negative future relationship scenarios involves reevaluation of outcomes.

Each block started with an instruction word (‘view’, ‘increase’, ‘decrease’) for 4 sec and consisted of 30 trials. Trial structure was: fixation cross for 900–1100 ms, picture for 3 sec, and blank screen for 2 sec. After each block, participants completed four ratings on a 1–5 scale: infatuation, attachment, valence, and arousal, and they also completed the PANAS at this moment [52]. After the regulation task, participants wrote down what they had thought to up- and down-regulate love feelings to verify that they had followed the instructions.

### EEG recording and preprocessing

The EEG was recorded using a 32-channel amplifier and data acquisition software (ActiveTwoSystem, BioSemi). The 32 Ag-AgCl active electrodes were placed upon the scalp by means of a head cap (BioSemi), according to the 10–20 International System. Vertical electro-oculogram and horizontal electro-oculogram were recorded by attaching additional electrodes (UltraFlat Active electrodes, BioSemi) above and below the left eye, and at the outer canthi of both eyes. Another two electrodes were attached to the left and right mastoids. An active electrode (common mode sense) and a passive electrode (driven right leg) were used to comprise a feedback loop for amplifier reference. All signals were digitized with a sampling rate of 512 Hz, a 24 bit A/D conversion and a low pass filter of 134 Hz. The EEG data were analyzed with BrainVision Analyzer 2 (Brain Products, Gilching, Germany). Per participant, a maximum of one bad electrode included in the analyses (see below) was corrected using spherical spline topographic interpolation. Offline, an average mastoids reference was applied and the data were filtered using a 0.1–30 Hz band pass filter (phase shift-free Butterworth filters; 24 dB/octave slope) and a 50 Hz notch filter. Data were segmented in epochs from 200 ms pre-stimulus until 3000 ms post-stimulus onset. Ocular artifact correction was applied semi-automatically according to the Gratton and Coles algorithm [54]. The 200 ms pre-stimulus period was used for baseline correction. Artifact rejection was performed at individual electrodes with the criterion minimum and maximum baseline-to-peak -75 to +75 μV. Because at least 12 trials are needed to adequately estimate emotion regulation effects in the LPP amplitude [55], the one participant that had fewer than 12 trials left per electrode per condition was excluded from the EEG analyses, as mentioned above. At the three electrodes used in the analyses (see below), the average number of accepted trials per condition ranged from 29.5 to 29.8 out of 30.

### Statistical analyses

Questionnaire scores were analyzed with independent samples *t*-tests to test for differences between groups (adjusted *t*, *df*, and *p* values are shown when the Levene’s test for equality of variances indicated variance differences between the groups). Besides the one-sample *t*-test against 5 (= neutral), the perceived control scores were analyzed with three ANOVAs: one ANOVA with factors Love type (love in general, infatuation, attachment) and Group (relationship, break-up), one ANOVA with factors Self/People (self, people in general) and Group, and one ANOVA with factors Intensity/Object (intensity, object) and Group. Pearson correlation coefficients were computed between the seven perceived control scores and the two ERQ subscales across groups. Ratings and PANAS scores after the view conditions were analyzed with an ANOVA with factors Picture (partner, neutral) and Group. Ratings and PANAS scores following the three conditions with partner pictures were analyzed with an ANOVA with factors Regulation (view, up-regulation, down-regulation) and Group. In this analysis, only significant effects involving the factor Regulation are reported, because the main effect of Group is not relevant for the research question.

Because the LPP begins in the time range of the classic P300 [19] and can last as long as the stimulus duration [56], the ERP was quantified by mean amplitude measures in four time windows based on previous work [47, 48, 56–58]: 300–400 ms, 400–700 ms, 700–1000 ms, 1000–3000 ms. For each time window, mean amplitudes measures at Fz, Cz, and Pz were subjected to two ANOVAs. The first concerned the two view blocks and tested the factors Picture, Group, and Caudality (Fz, Cz, Pz). Only significant effects involving the factor Picture are reported, because those are relevant for the research question. The second ANOVA concerned the three blocks with partner pictures and tested the factors Regulation, Group, and Caudality. In this analysis, only significant effects involving the factor Regulation are reported, because those are relevant for the research question. When applicable, the degrees of freedom were corrected using the Greenhouse-Geisser correction. The *F* values, uncorrected degrees of freedom, the ε values and corrected probability values are reported. A significance level of 5% (two-sided) was selected and Fisher’s least significance difference (LSD) procedure was applied. This procedure controls type I error rate by conducting follow-up tests for significant main and interaction effects only. Those follow-up tests were paired samples *t*-tests testing differences between conditions across both groups (in case of significant main or interaction effects without the factor Group) or within groups (in case of significant interactions with the factor Group).

## Study 2 –Results

### Group characteristics

All participants had an opposite-sex partner. The average time since the break-up was 3.0 months (range = 0.5–13.5). Ten of these break-ups were initiated by the partner, six by the participant, and four break-ups were a joint decision. See Table 4 for the other group characteristics and the statistics related to group differences. The relationship and break-up groups did not differ in how long they had known their partner for, how long ago their love feelings had started, and the duration of their relationships. The break-up groups did tend to report lower relationship quality than the relationship group. The break-up group also felt less attached and tended to feel more infatuated with their partner than the relationship group. Moreover, the break-up group tended to have experienced less positive affect during the past two weeks and had experienced more negative affect in the past two weeks and at the start of the testing session than the relationship group. Finally, the relationship and break-up groups did not differ in their habitual use of reappraisal and suppression. Thus, the two groups differed from each other on variables that can be expected to be related to whether someone is in a relationship or has experienced a break-up, but the groups did not differ on variables that should be unrelated to relationship status.

### Perceived control

The mean score on the 17 perceived control questions was 4.5 (*SD* = 1.4). A one-sample *t*-test showed that this was significantly lower than 5 (= neutral), *t*(39) = -2.2, *p* = .032, which suggests that participants perceive love feelings as uncontrollable. See Table 3 for the mean perceived control over the seven different aspects of love. There was a significant main effect of Love type, *F*(2,76) = 19.8, ε = 1.0, *p* < .001. Participants felt more in control of feelings of attachment than of feelings of infatuation or love in general, both *p*s < .001. In addition, there tended to be a main effect of Self/People, *F*(1,38) = 3.9, *p* = .056. Participants tended to feel that they were less able to control their love feelings than people in general are. Finally, there was a main effect of Intensity/Object, *F*(1,38) = 14.6, *p* < .001. Participants felt more in control of the intensity than of the object of their love feelings. In none of these analyses, the main effect of Group or interactions with Group were significant, all *F*s < 2.4, all *p*s > .13, so perceived control over different aspects of love feelings did not differ between the relationship and break-up groups.

**Fig 2:**
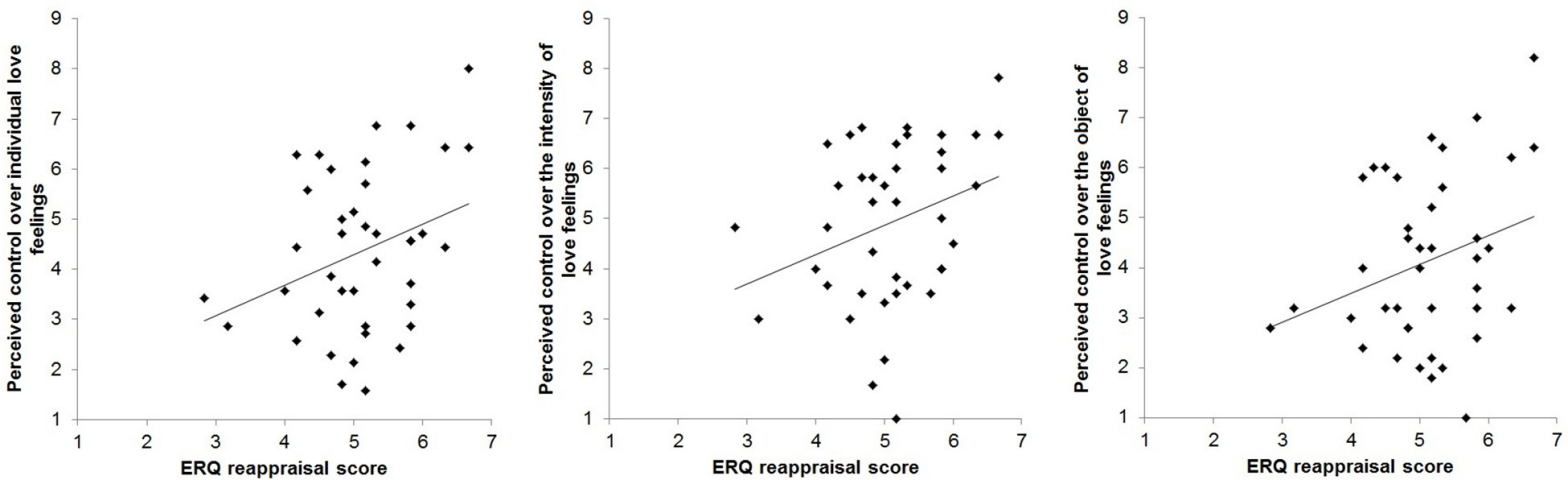
Scatterplots displaying the positive correlations between the ERQ reappraisal score and perceived control over various aspects of love feelings.

The ERQ reappraisal score correlated positively with perceived control over individual love feelings, *r*(38) = .32, *p* = .044, and tended to correlate positively with perceived control over the intensity of love feelings, *r*(38) = .30, *p* = .056, and with perceived control over the object of love feelings, *r*(38) = .29, *p* = .075, see Fig 2. These findings suggest that the more participants used the reappraisal strategy to regulate emotions in their daily life, the more they perceived love feelings as controllable.

### Strategies

See Tables 1 and 2 for the regulation strategies reported. Participants mostly used distraction and reappraisal when heartbroken. Distraction was used more to feel better, while reappraisal by focusing on the negative aspects of the beloved/relationship was used more to decrease love feelings. The other strategies, such as reappraisal by thinking about the positive aspects of the situation, other ways of reappraising, avoidance, suppression (“making yourself strong (pretend) for the outside world”), eating/smoking, and expressing emotions were used least often when heartbroken. None of the participants reported the use of reappraisal by thinking about negative future scenarios. Two participants reported that they could not decrease love feelings. The use of the different strategies did not appear to differ between the relationship and break-up groups. Seeking social support was less popular in the current Dutch sample than in the US sample in Study 1.

Participants stressed the importance of communication/honesty and undertaking (new) activities with their beloved during long-term relationships. Communication/honesty was mostly used for maintaining long-term relationships. The break-up group used undertaking (new) activities with the beloved mostly to prevent love feelings from declining, while the relationship group used this strategy both to maintain their relationship and to prevent love feelings from declining. Strategies such as expressing love feelings to the beloved, spending (quality) time with the beloved, and loving unconditionally/making compromises were mentioned by some participants. The latter was used more for maintaining long-term relationships than for preventing love feelings from declining. Both reappraisal strategies (i.e., focusing on positive aspects of the beloved/relationship and thinking about positive future scenarios), trust, and spending time apart from the beloved were mentioned by some participants. Two participants specifically stated that love feelings would not decline if the relationship was good and/or that they would end the relationship if love feelings would decline. Other than the above-mentioned difference in the context of undertaking (new) activities, there were no obvious differences between the relationship and break-up groups. There were no major differences between this Dutch sample and the US sample in Study 1.

To conclude, participants reported to use several behavioral and cognitive strategies in heartbreak and long-term relationship contexts. As in Study 1, some of these strategies were the typical cognitive and behavioral emotion regulation strategies, such as reappraisal, distraction, situation selection, and suppression. As in Study 1, some strategies seemed specific for feeling better during heartbreak (i.e., emotion regulation) or for maintaining long-term relationships, while strategies such as reappraisal by focusing on the negative aspects of the beloved or the relationship and undertaking (new) activities with the beloved were used to down- and up-regulate love feelings, respectively.

### Ratings

**Fig 3:**
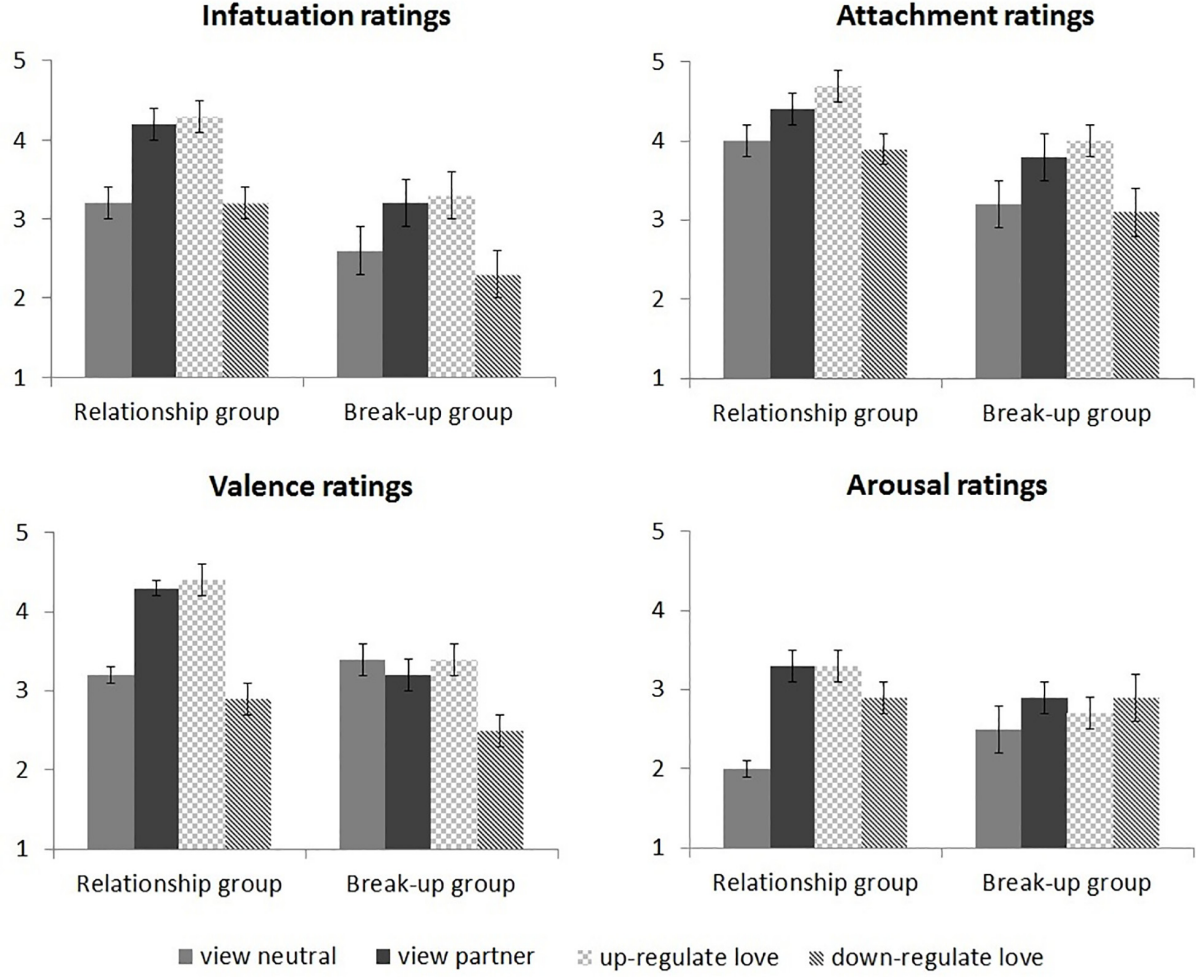
Infatuation, attachment, valence, and arousal ratings after each block.

See Fig 3 for the infatuation, attachment, valence, and arousal ratings at the end of each block in the regulation task.

#### View blocks

The infatuation ratings after the two view blocks showed main effects of Picture, *F*(1,38) = 27.1, *p* < .001, and Group, *F*(1,38) = 5.6, *p* = .023. Infatuation was higher after passive viewing of partner than neutral pictures, and higher in the relationship than the break-up group. Attachment ratings also showed main effects of Picture, *F*(1,38) = 23.9, *p* < .001, and Group, *F*(1,38) = 5.6, *p* = .023. Attachment was higher after passive viewing of partner than neutral pictures, and higher in the relationship than the break-up group. Thus, partner pictures elicited more feelings of infatuation and attachment than neutral pictures in both groups, which shows that the use of partner pictures was effective in eliciting love feelings [38].

For valence ratings, the main effects of Picture, *F*(1,38) = 9.9, *p* = .003, and Group, *F*(1,38) = 7.7, *p* = .008 were modulated by a significant Picture x Group interaction, *F*(1,38) = 20.7, *p* < .001. The relationship group felt more positive after passive viewing of partner than neutral pictures, *p* < .001, whereas the break-up group did not, *p* = .41. Arousal ratings showed a main effect of Picture, *F*(1,38) = 26.8, *p* < .001, which was modulated by a significant Picture x Group interaction, *F*(1,38) = 7.9, *p* = .008. The relationship group felt more aroused after passive viewing of partner than neutral pictures, *p* < .001, whereas the break-up group did not, *p* = .10. Thus, partner pictures elicited positive and arousing feelings in the relationship group but not in break-up group, which confirms that the two groups differed in anticipated ways.

#### Regulation blocks

The infatuation ratings after the three blocks with partner pictures showed a main effect of Regulation, *F*(2,76) = 39.6, ε = .84, *p* < .001. Infatuation was lower after down-regulation than after passive viewing or up-regulation, both *p*s < .001. Attachment ratings also showed a main effect of Regulation, *F*(2,76) = 36.7, ε = .91, *p* < .001. Attachment was highest after up-regulation, intermediate after passive viewing, and lowest after down-regulation, all *p*s < .013. Valence ratings showed a main effect of Regulation, *F*(2,76) = 31.6, ε = .98, *p* < .001. Participants felt less positive after down-regulation than after passive viewing or up-regulation, both *p*s < .001. Arousal ratings showed no significant effects involving the factor Regulation, all *p*s > .26. To summarize, subjective love feelings were modulated in the expected directions by instructed love regulation in both groups. Up-regulation of love increased feelings of attachment, and down-regulation of love decreased feelings of infatuation and attachment. Finally, down-regulation of love decreased the pleasantness of feelings in both groups.

### Positive and negative affect

**Fig 4:**
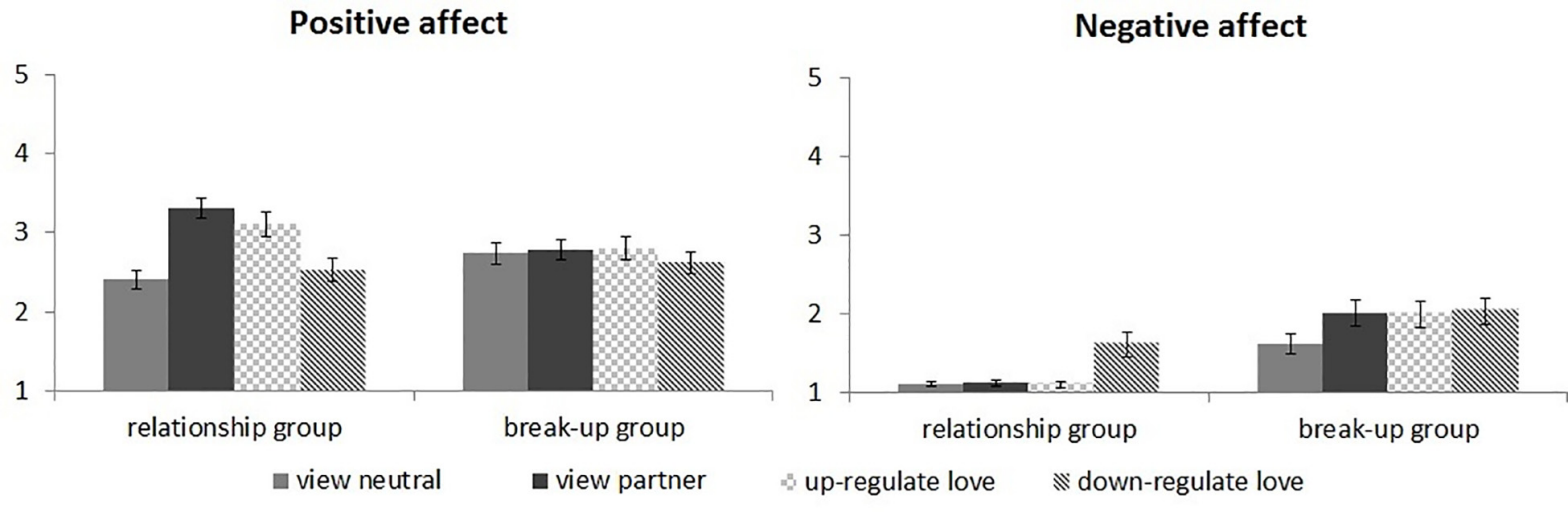
Positive and negative affect after each block, separately for both groups.

See Fig 4 for positive and negative affect during the regulation task.

#### View blocks

Positive affect after the two view blocks showed a main effect of Picture, *F*(1,38) = 27.2, *p* < .001, which was modulated by a significant Picture x Group interaction, *F*(1,38) = 21.7, *p* < .001. The relationship group experienced more positive affect after passive viewing of partner than neutral pictures, *p* < .001, while the break-up group did not, *p* = .71. Negative affect showed main effects of Picture, *F*(1,38) = 9.6, *p* = .004, and Group, *F*(1,38) = 25.7, *p* < .001, which were modulated by a significant Picture x Group interaction, *F*(1,38) = 8.7, *p* = .005. The break-up group experienced more negative affect after passive viewing of partner than neutral pictures, *p* = .006, while the relationship group did not, *p* = .69. In short, partner pictures elicited positive affect in the relationship group, but negative affect in the break-up group, again confirming that the groups differed in expected ways.

#### Regulation blocks

Positive affect after the three blocks with partner pictures showed a main effect of Regulation, *F*(2,76) = 21.3, ε = .94, *p* < .001, which was modulated by a significant Regulation x Group interaction, *F*(2,76) = 8.4, ε = .94, *p* = .001. While the relationship group experienced most positive affect after passive viewing of partner pictures, intermediate positive affect after up-regulation and least positive affect after down-regulation, all *p*s < .034, positive affect in the break-up group was not affected by instructed love regulation, all *p*s > .17. Negative affect showed a main effect of Regulation, *F*(2,76) = 6.4, ε = .74, *p* = .007, which was modulated by a significant Regulation x Group interaction, *F*(2,76) = 5.0, ε = .74, *p* = .017. The relationship group experienced most negative affect after down-regulation, both *p*s < .008, whereas negative affect in the break-up group was not affected by instructed love regulation, all *p*s > .67. To summarize, love regulation decreased positive affect in the relationship group, although down-regulation decreased positive affect more than up-regulation. Down-regulation also increased negative affect in the relationship group. Regulation of love feelings did not influence affect in the break-up group.

### Event-related potentials

#### View blocks

**Fig 5:**
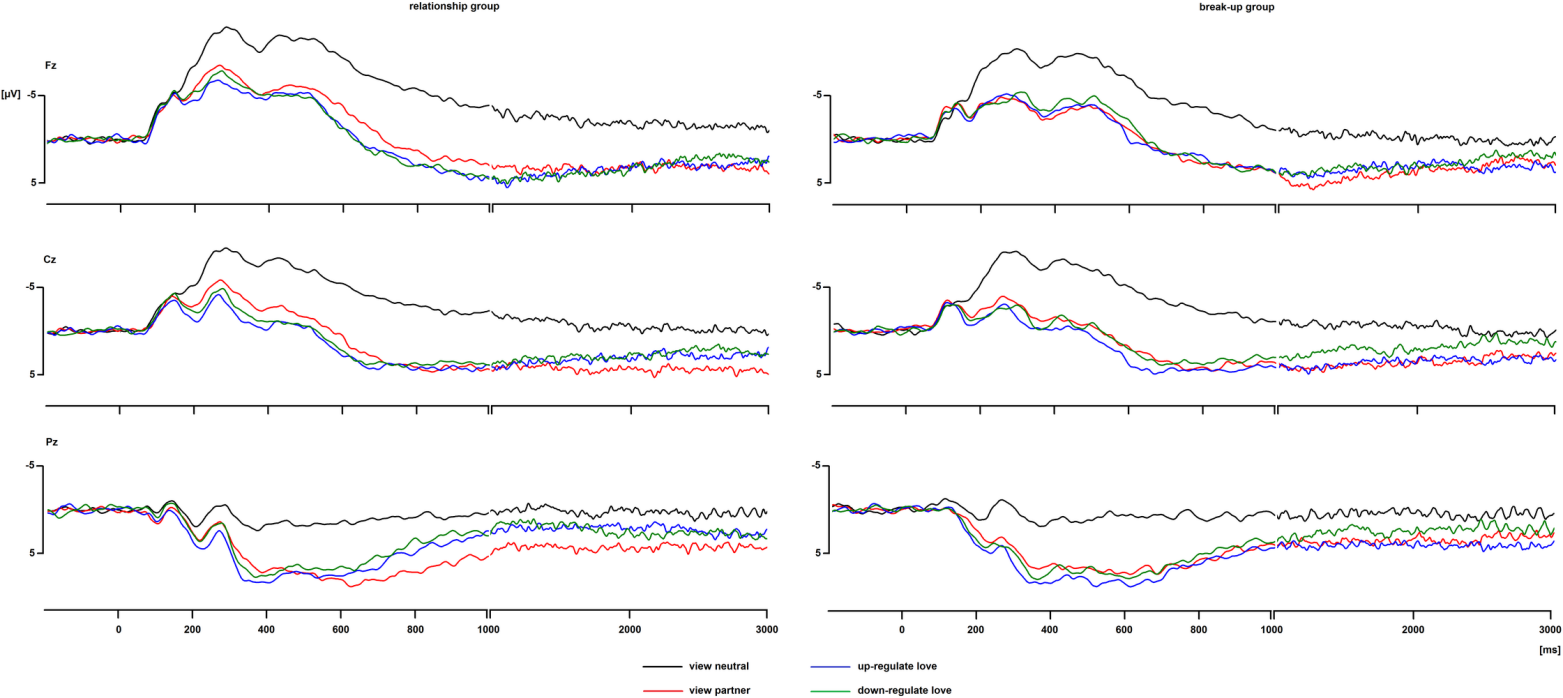
ERP waveforms at Fz, Cz, and Pz for the four conditions, for each group separately.

**Fig 6:**
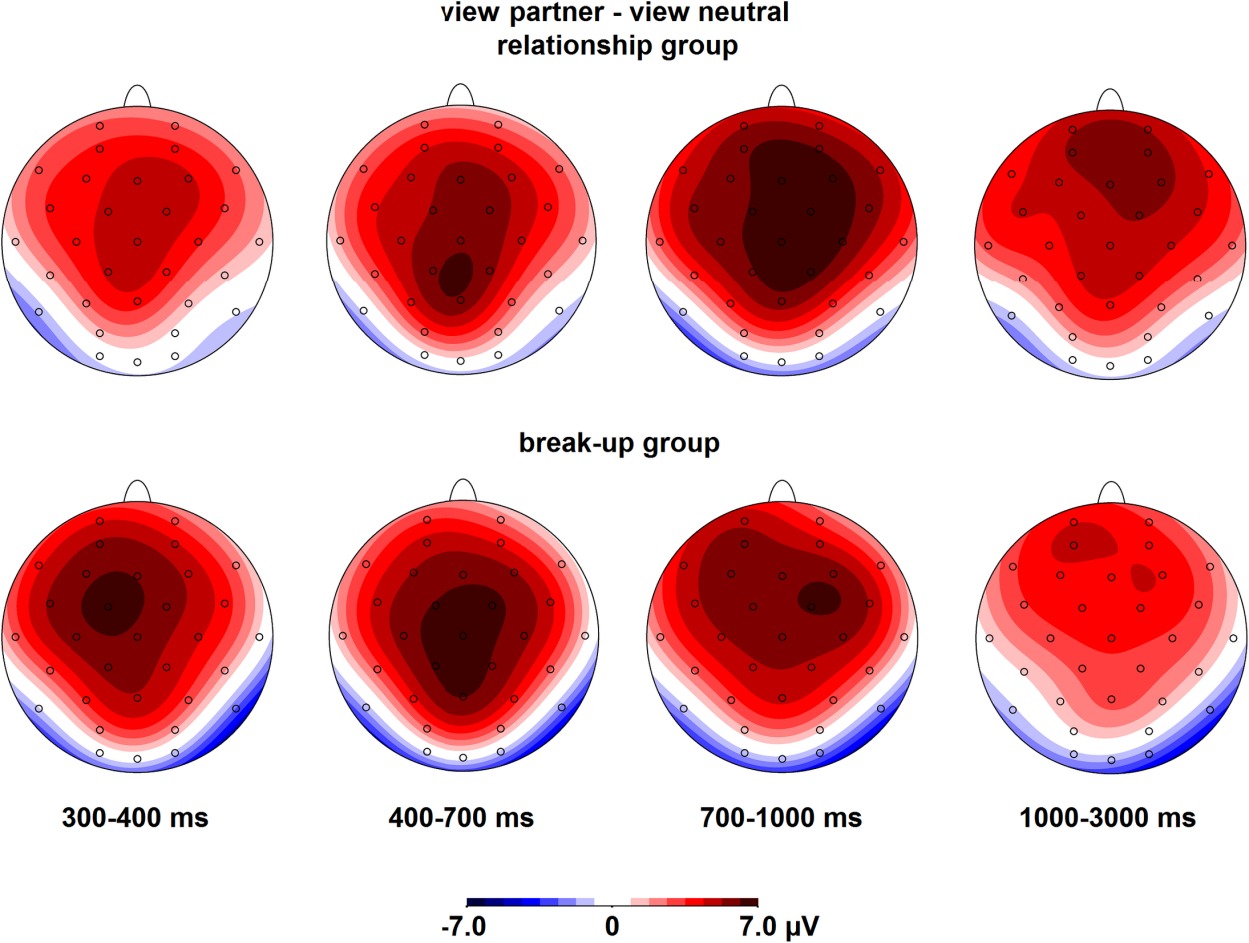
Scalp topographies of the differences between passive viewing of partner and neutral pictures, separately for both groups.

See Fig 5 for the ERP waveforms and Fig 6 for the scalp topographies of the differences between partner and neutral pictures in the view blocks. In all four time windows, there was a significant main effect of Picture (300–400 ms: *F*(1,34) = 72.6, *p* < .001, 400–700 ms: *F*(1,34) = 101.6, *p* < .001, 700–1000 ms: *F*(1,34) = 112.3, *p* < .001 and 1000–3000 ms: *F*(1,34) = 51.1, *p* < .001), indicating that the ERP between 300–3000 ms was more positive in response to the partner than neutral pictures. The interactions involving the factors Picture and Group were not significant in any of the time windows, all *F*s < 1, *ns*, which indicated that the ERP response to the partner compared to the neutral pictures did not differ between the relationship and break-up groups. Partner and neutral pictures differ in multiple ways: partner pictures were familiar to the participants, elicited emotional feelings, displayed at least one familiar person, and may have displayed the participant him-/herself. Because all of these factors modulate the ERP [32, 42, 59–61], it is not surprising that the ERP difference between partner and neutral pictures is so extended in time and topography, and similar between the two groups. Please note that the up- and down-regulation effects of interest discussed below involve comparisons between up- and down-regulation of responses to the partner pictures only.

#### Regulation blocks

**Fig 7:**
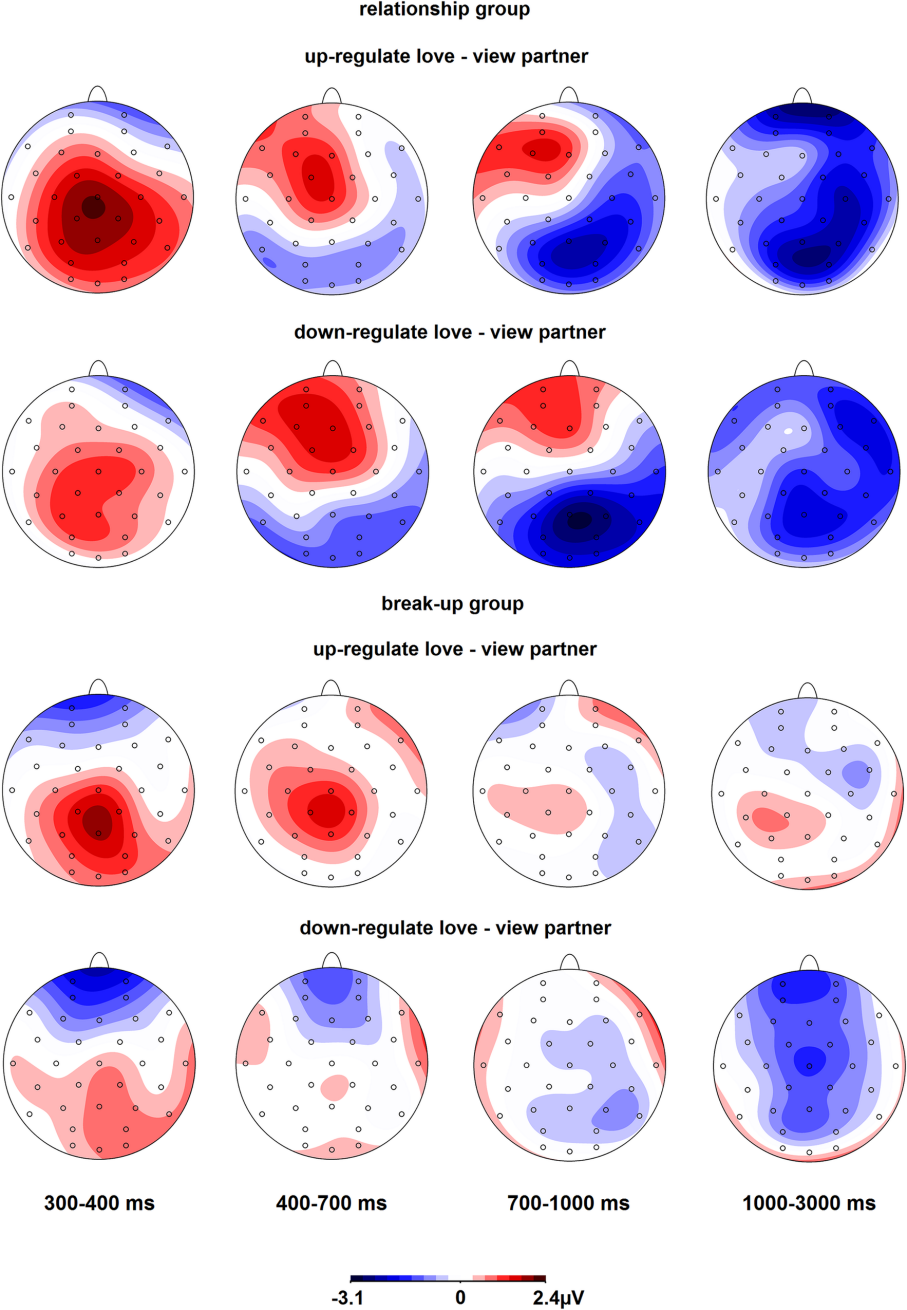
Scalp topographies of the differences between regulation and passive viewing of partner pictures, separately for both groups.

See Fig 5 for the ERP waveforms and Fig 7 for the scalp topographies of the differences between regulation and view conditions. In the 300–400 ms time window, there were a main effect of Regulation, *F*(2,68) = 4.4, ε = .96, *p* = .018, and a Regulation x Caudality interaction, *F*(4,136) = 3.7, ε = .62, *p* = .020. The ERP was more positive for up-regulation than passive viewing at Cz and Pz, both *p*s < .004. In the 400–700 ms time window, there was a Regulation x Group x Caudality interaction, *F*(4,136) = 4.9, ε = .72, *p* = .004, but none of the post hoc tests were significant. In the 700–1000 ms, there were Regulation x Caudality, *F*(4,136) = 5.8, ε = .65, *p* = .002, and Regulation x Group x Caudality, *F*(4,136) = 5.2, ε = .65, *p* = .004, interactions. In the relationship group, the ERP was less positive for up- and down-regulation than passive viewing at Pz, both *p*s < .002. In the break-up group, none of the post hoc tests were significant. In the 1000–3000 ms time window, there was a Regulation x Group x Caudality interaction, *F*(4,136) = 2.7, ε = .78, *p* = .048. In the relationship group, the ERP was less positive for down-regulation than passive viewing at Cz and Pz, both *p*s < .041, and for up-regulation than passive viewing at Pz, *p* = .005. In the break-up group, none of the post hoc tests were significant. Inspection of the data revealed that even though the break-up group showed a less positive ERP for down-regulation at Cz (and at Pz to a lesser extent), the variation in ERP amplitudes was larger in the break-up group (down-regulation effect at Cz = -1.7 μV, *SD* = 4.0) than the relationship group (down-regulation effect at Cz = -1.6 μV, *SD* = 3.0), which explains why the effect did not reach significance in the break-up group.

**Fig 8:**
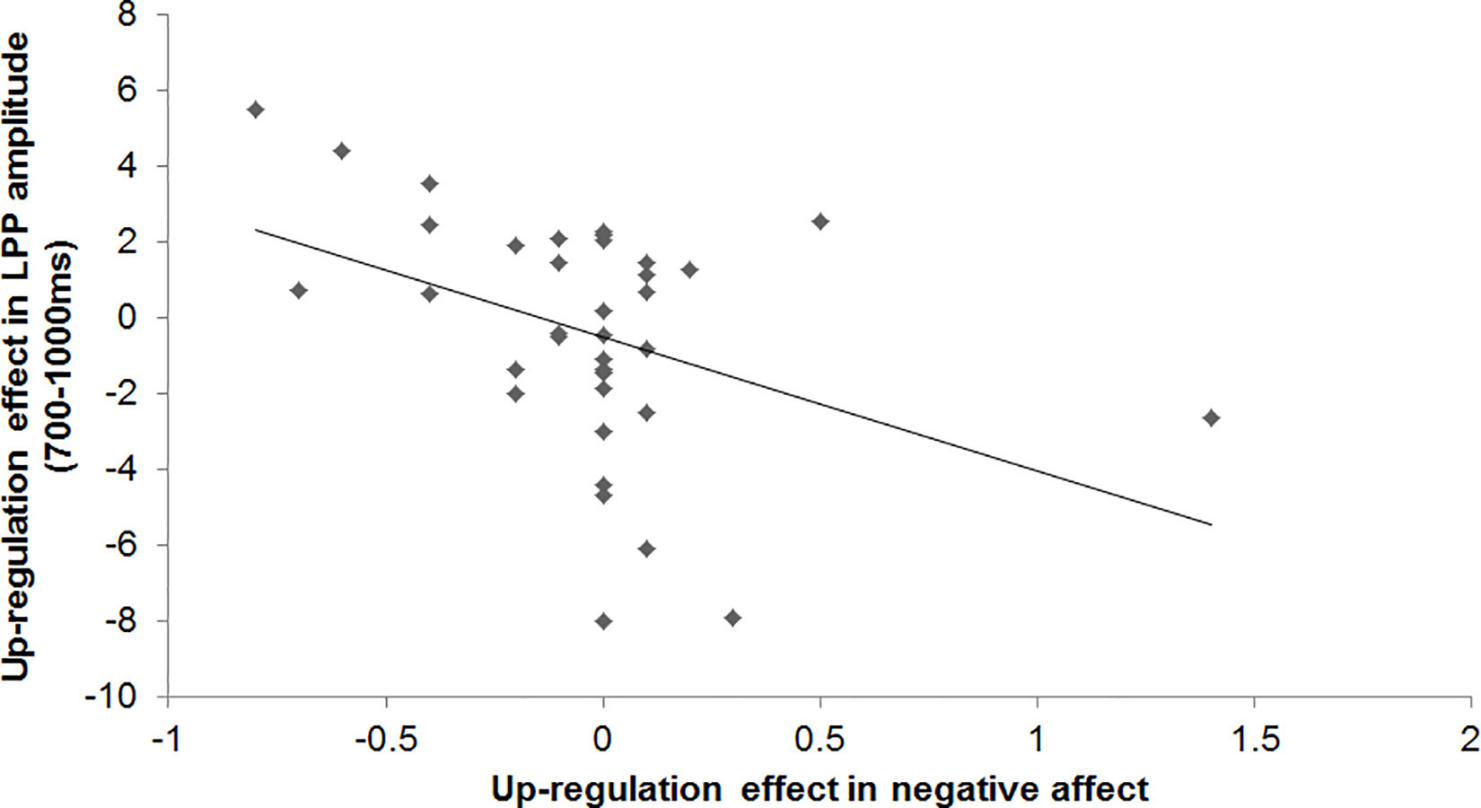
Scatterplot displaying the negative correlation between the up-regulation effect in the LPP amplitude (averaged across electrodes Cz and Pz) between 700–1000 ms and the up-regulation effect in negative affect. A negative up-regulation effect in negative affect means a reduction in negative affect due to love up-regulation. A positive up-regulation effect in the LPP amplitude means that the LPP was enhanced for love up-regulation.

To explore any associations between the LPP amplitude and self-reports measures, Pearson correlation coefficients were computed between regulation effects in the LPP amplitude and regulation effects in infatuation ratings, attachment ratings, valence ratings, arousal ratings, positive affect, and negative affect, across groups. Because the regulation effects were largest at electrodes Cz and/or Pz, LPP regulation effects were averaged across these two electrodes. In the 700–1000 ms time window, the up-regulation effect in the LPP amplitude was negatively correlated with the up-regulation effect in negative affect, *r*(34) = -.40, *p* = .015, see Fig 8. This correlation was not inflated by the possibly outlying data point (i.e., up-regulation effect in negative affect = 1.5), because the correlation was even greater and more significant after exclusion of this data point, *r*(33) = -.44, *p* = .008. As can be seen in Fig 8, the more participants showed an enhanced LPP in response to up-regulation compared to passive viewing between 700–1000 ms, the more their negative affect decreased as a result of love up-regulation. The other correlations between the up-regulation effects in the LPP amplitude in any of the time windows and the up-regulation effects in self-reports were not significant, -.31 < all *r*s(34) < .32, all *p*s > .063. None of the down-regulation effects in the LPP amplitude in any of the time windows were significantly correlated with down-regulation effects in self-reports, -.20 < all *r*s(34) < .24, all *p*s > .17.

To summarize, up-regulation elicited a more positive ERP than passive viewing at midline centro-parietal electrodes between 300–400 ms. In addition, up- and down-regulation elicited a less positive ERP than passive viewing mostly at midline parietal electrodes between 700–3000 ms in the relationship group. The more love up-regulation enhanced the LPP amplitude between 700–1000 ms, the greater the decrease in negative affect by love up-regulation.

## Discussion

Because love feelings may be more or less intense than desired, it would be helpful if people could up- and down-regulate feelings of romantic love at will. In two studies, we examined preconceptions about, strategies for, and the feasibility of love regulation.

As expected, participants had the preconception that love is somewhat uncontrollable, as indicated by their scores on the series of questions assessing the perceived controllability of love feelings. Moreover, a few participants reported that they are unable to decrease love feelings when heartbroken. Some participants even stated that love feelings should not be up-regulated to maintain long-term relationships, because declining love feelings would indicate that the relationship is not meant to be. Research, however, has shown that infatuation (i.e., passionate love) and attachment (i.e., companionate love) typically do decline over time [14, 16], so having this opinion might limit one’s chances of having long-lasting relationships. However, the mean score on the perceived control questions approached the midpoint of the scale, indicating that participants did not entirely reject the idea of controllable love. In addition, participants perceived some aspects of love to be more controllable than other aspects. Participants perceived feelings of attachment as more controllable than feelings of infatuation and they felt more in control of the intensity of love feelings than of who they are in love with. Finally, the more participants use the reappraisal strategy to regulate emotions in their daily life, the more they perceived love feelings as controllable, which provides a hint that reappraisal may be an effective love regulation strategy. Nevertheless, the questions about perceived control over love feelings have a couple of limitations. First, the two studies were conducted in different countries, in different languages, and with relatively small samples with different gender ratios. Second, in order to not be too statistically conservative in this explorative study, we did not correct for the number of statistical tests employed. Please note that the tests we performed are not independent, which reduces the chance of type I errors [62]. In addition, we used two-sided tests even when we had an a priori directional hypothesis. This was done to not increase the chance of Type I errors and to not exclude the possibility of observing any effects that were contrary to the hypothesis, again, because of the exploratory nature of the study. Finally, to the extent that the measures in Studies 1 and 2 overlapped, we base the conclusions above only on findings that replicated in both studies. This greatly reduces the chance that our conclusions are based on country-, language-, or gender-specific effects, or on spurious findings. It would be interesting to test whether perceived control over love feelings varies between regulation directions (i.e., whether people think it is easier to down-regulate love than to up-regulate love, or vice versa) in future studies.

We asked participants what they typically do or think when they are heartbroken and when maintaining long-term relationships. Participants reported the use of prototypical emotion regulation strategies such as reappraisal, distraction, and situation selection. Only one participant mentioned using suppression. Research has shown that expression suppression does not actually alter the intensity of feelings and that it has negative effects on cognitive and social functioning [21], so it might not be an adaptive strategy for regulating love feelings.

Importantly, responses to the questions suggested that there was a dissociation in the use of certain strategies for regulating actual love feelings (i.e., love regulation) versus feeling better during heartbreak (i.e., emotion regulation) or maintaining long-term relationships. In the context of heartbreak, reappraisal was often used, especially to decrease love feelings rather than to feel better. In contrast, distraction was used during heartbreak more to feel better than to decrease love feelings. It has been shown that people prefer to use distraction over reappraisal in situations in which emotions are very intense [63], which is often the case during heartbreak. Reappraisal may be more advantageous in the long run though, because decreasing love feelings might help people to move on after a break-up.

Some participants reported avoiding beloved-related cues, such as pictures or conversations, when heartbroken, which is a situation selection strategy [21]. It has been proposed that romantic love shows parallels to drug addiction [2, 64]. Beloved-related cues elicit love feelings [38], just like drug-related cues increase drug craving [65], so avoiding beloved-related cues may reduce ‘craving’ for the beloved in the short term. However, one type of treatment for substance dependence and other mental disorders is exposure therapy, which is based on the mechanism of extinction [66]. Because avoidance of beloved-related cues might prevent extinction of the love feelings, it may not be a suitable strategy for down-regulating love feelings in the longer term.

In the context of long-term relationships, participants often mentioned the importance of communication/honesty and of undertaking (new) activities with their beloved. While communication/honesty was used more to maintain long-term relationships than to prevent love from declining, undertaking (new) activities with the beloved was mostly used to prevent love from declining. Previous work suggests that doing exciting things with the beloved may indeed be a successful strategy for love up-regulation [67, 68]. Correspondingly, research on long-term romantic love has shown that married couples who engaged in novel and challenging activities together reported increases in love, closeness, and relationship quality [69, 70]. Thus, undertaking novel and exciting activities with the beloved, which is a situation selection strategy [21], may be an effective behavioral strategy for up-regulating love feelings. Surprisingly, reappraisal was mentioned only infrequently in the context of maintaining long-term relationships. Given that reappraisal is an effective and healthy emotion regulation strategy [21, 22], it may be an adaptive strategy to prevent love feelings from declining in long-term relationships.

The four open questions about the use of behavioral and cognitive strategies in the contexts of heartbreak and long-term relationships have some limitations. First, these data were analyzed qualitatively rather than quantitatively. Second, the other questionnaires and tasks used in both studies restricted the samples to participants who were in love (Study 1), who were in a romantic relationship, or who had recently experienced a romantic break-up (Study 2). Therefore, participants will have answered questions that did not match their current status (e.g., answering questions about heartbreak while in a happy relationship) or prior experience (i.e., some participants may have never been heartbroken or in a long-term relationship, in which case they replied what they think they would do in those circumstances). It is important to note that these four strategy questions were used more to explore what types of strategies people employ in their love life to aid the design of future studies on love regulation rather than to provide a stringent test of a priori hypotheses. Nevertheless, the current findings await confirmation in future studies with quantitative analyses and matching of questions with prior experience and/or current status.

In the four open strategy questions, we did not ask participants about the effectiveness of the strategies they listed. In Study 2, in contrast, we did assess the effectiveness of explicit love up- and down-regulation using the cognitive reappraisal strategy. We measured regulation success by asking participants how much infatuation and attachment they experienced after each regulation condition, because self-report is the only way to assess phenomenological experience [39]. When instructed to up-regulate love feelings by thinking about positive aspects of the partner or the relationship or imagining positive future scenarios, participants reported increased levels of attachment. Although love up-regulation numerically increased feelings of infatuation, this effect was not statistically significant. Thus, love up-regulation using reappraisal may be more successful for up-regulating attachment than infatuation. Future research could test whether other strategies may be more effective for up-regulating infatuations levels. Because long-term relationships are threatened by diminishing levels of infatuation and attachment over time [14, 16], up-regulation of love feelings might help to stabilize long-term relationships. Although it has been shown before that people idealize their beloved [32, 33] and that partner idealization is associated with greater relationship satisfaction [34], the current study is unique in showing that people are capable of up-regulating their love feelings deliberately and intentionally.

When instructed to down-regulate love feelings by thinking about negative aspects of the partner or the relationship or imagining negative future scenarios, participants reported decreased levels of infatuation and attachment, as expected. This has important implications for people whose love feelings are stronger than desired. For example, this finding suggests that after the dissolution of a long-term relationship, when levels of attachments are presumably higher than levels of infatuation [14], love regulation using reappraisal may be used to cope with the break-up by decreasing feelings of attachment. In addition, the current findings suggest that love down-regulation using reappraisal may be used to decrease feelings of infatuation, for example when early stage love feelings are unreciprocated or when someone develops a crush on someone else than their partner. Although previous studies have shown that people can implicitly derogate the attractiveness of people other than the current partner [71, 72], the current investigation is unique because it reveals that people can deliberately down-regulate their love feelings for their (ex-)partner.

Because self-reports are the only way to assess subjective feelings [39], they are often used in behavioral and neuroimaging studies on emotion regulation as a way to assess regulation success (e.g., [49, 57, 73, 74]). However, self-reports do suffer from desirability biases and demand characteristics [40, 41]. Participants were not informed of the exact research purpose or hypothesis before testing, but they were instructed to increase or decrease their love feelings using cognitive reappraisal. So when they were asked to rate infatuation and attachment levels at the end of each block, their responses may have been biased by their perception of the study’s hypothesis. Still, the instructions mentioned ‘love feelings’ whereas the ratings mentioned ‘infatuation’ and ‘attachment’, which may have made our expectations a little less obvious. Also, even though we did not have different expectations about the feasibility of increasing and decreasing love, and participants had no reason to assume we had, the up-regulation effects in self-reported infatuation and attachment were numerically smaller (0.1 to 0.3 points on a 1–5 scale) than the down-regulation effects (0.5 to 1.0 points on a 1–5 scale), which makes it less likely that participants responded according to a perceived hypothesis rather than according to their feelings. Nevertheless, the current results await replication in studies in which the hypothesis would be more obscure to participants. This could for example be established by instructing participants to think about positive/negative aspects or future scenarios without mentioning that this is supposed to change the intensity of their love feelings. Also, note that the self-reports regarding valence, arousal, positive affect, and negative affect discussed next are less susceptible to demand characteristics since participants were not instructed to change how positive, negative, or aroused they felt.

Because it is important to dissociate the concept of love regulation from the well-established concept of emotion regulation, we asked participants how negative or positive they felt after each regulation condition. Participants who were in a romantic relationship with their beloved experienced more unpleasant feelings, less positive affect, and more negative affect following love down-regulation. This was expected, as down-regulation of love feelings for a current long-term partner is usually undesirable. However, also participants who had recently experienced a break-up unexpectedly experienced more unpleasant feelings after love down-regulation. It may be that love down-regulation by focusing on negative aspects of the partner or the relationship or imagining negative future scenarios makes people feel bad because it involves negative thoughts. Although the current study did not study the long-term effects of love down-regulation using reappraisal, it has recently been shown that thinking negative thoughts about the relationship has adaptive features when recovering from a romantic break-up [75]. So, it is important to investigate both the short- and the long-term effects of love regulation, as those may be dissociated.

Love up-regulation resulted in decreased positive affect in participants who were in a relationship, which was unexpected. This may have occurred because of the effort it takes to apply cognitive reappraisal [18]. It could be that people who are in a happy relationship (as indicated by self-reported relationship quality) may prefer to just look at their partner, rather than to have to come up with positive aspects of the partner or the relationship, or positive future scenarios on demand. We did not test the long-term effects of love regulation, but it might be that even though it may be cumbersome at this moment to use reappraisal to up-regulate love feelings, it may have beneficial long-term effects in the context of romantic relationships. Future studies are needed to replicate this unexpected effect, to determine why it occurs, to explore if and how it can be reduced, and to test if it is perhaps accompanied by an advantageous long-term effect. Even though love up-regulation resulting in decreased positive affect in participants who were in a relationship is in contrast to the hypothesis, it does show that participants were not just regulating their emotions. In that case, love up-regulation would have resulted in more positive feelings in both groups. This suggests that it is important to distinguish between the effects of love regulation on love feelings and on affect, as a desired effect in love feelings might not result in better affect in the short run.

Love regulation did not change subjective arousal (cf. [76]. It could be that the five-point rating scale we used was too coarse to detect any changes in arousal due to love regulation. It may be better to use a finer scale in future studies. Alternatively or additionally, recent work has shown that reappraising anxiety as excitement (i.e., changing the valence from negative to positive) improved performance in anxiety-provoking tasks compared to trying to calm down (i.e., reducing arousal) [77], so it may be more beneficial to change valence rather than arousal when regulating love feelings. More research is needed to test this suggestion.

Unlike the self-reported infatuation and attachment levels, the LPP amplitude is not a direct measure of love intensity. The advantage of the LPP amplitude over self-reported feelings is that it is not susceptible to social desirability biases and demand characteristics. Because the LPP amplitude is typically enhanced in response to both positive and negative stimuli, the LPP does not reflect whether a stimulus elicits positive or negative feelings. Instead, the LPP amplitude has been used as an objective measure of regulation success [19] because it reflects the affective and motivational significance of a stimulus and the resulting motivated attention instead [42]. So, the LPP amplitude in response to a picture of the partner indicates how emotionally or motivationally significant the partner is and how much attention is being paid to him/her. The instruction to up-regulate love feelings resulted in a more positive ERP between 300–400 ms (cf. [27]. The latency and the midline centroparietal topography of this regulation effect confirms that regulation instructions modulated the LPP component [42]. The enhanced LPP indicates that love up-regulation enhances the affective and motivational significance of, and the resulting motivated attention to the (ex-)partner. Because stronger love feelings would result in enhanced significance of the partner, the enhanced LPP with love up-regulation corroborates the self-report finding that people are able to up-regulate their love feelings deliberately.

Love down-regulation decreased the LPP amplitude between 700–3000 ms in participants who were in a romantic relationship, which indicates that love down-regulation reduced the affective and motivational significance of, and the resulting motivated attention to the partner. Because weaker love feelings would result in reduced significance of the partner, the reduced LPP with love down-regulation corroborates the self-report finding that people are able to down-regulate their love feelings deliberately. It is important to note that the ERP reflects brain activation elicited by events, which are the presentations of partner and neutral pictures in this case. A reduced LPP amplitude by down-regulation is therefore not at odds with the increased self-reported negative affect at the end of the down-regulation block. That is, a reduced affective and motivational significance of, and motivated attention to the partner pictures (as reflected by the LPP amplitude) may very well be accompanied by an increase in general negative affect that is not linked to the 3-sec presentation of a picture and will therefore not be reflected in the ERP (e.g., because the baseline correction removed the effect). It is interesting that the down-regulation effect occurred a few hundred milliseconds later than the up-regulation effect (cf. [78]), which suggests that love down-regulation takes more time to take effect than love up-regulation. The down-regulation effect in the LPP amplitude did not reach significance in participants that had experienced a break-up, which is ironic because love down-regulation might benefit them more than people who are in a happy relationship. Greater interindividual variation is the likely cause of the down-regulation effect not being significant in the break-up group. This variation may have been due to the break-up group being rather heterogeneous in terms of time since break-up, intensity of love feelings for the ex-partner, and levels of positive and negative affect, since factors like these may affect love down-regulation success.

In contrast to the hypotheses, and to the notion that the LPP amplitude is modulated by regulation instruction according to the regulatory goal [19], the LPP amplitude was numerically, but not significantly, enhanced for down-regulation between 300–400 ms in both groups, and significantly reduced for up-regulation between 700–3000 ms in participants who were in a romantic relationship. Interpreting the LPP amplitude as reflecting the affective and motivational significance of, and the resulting motivated attention to a stimulus [42], the significantly reduced LPP amplitude for up-regulation between 700–3000 ms in participants who were in a romantic relationship suggests that, even though up-regulation initially (i.e., between 300–400 ms) increases the significance of, and attention to a current partner, it reduces it eventually (i.e., after 700 ms). Interestingly, the up-regulation effect in the LPP amplitude between 700–1000 ms showed significant individual differences. Even though the LPP was reduced by love up-regulation at the group level, participants who actually showed a more enhanced LPP amplitude as a result of love up-regulation in this time window also showed a greater decrease in negative affect as a result of love up-regulation. Because correlation does not imply causation, this effect could be interpreted in several ways. It might be that love up-regulation only leads to a reduction in negative affect when it is successful (as indicated by an enhanced LPP). Future research will need to replicate this effect and clarify its interpretation.

The unexpected LPP findings challenge the interpretation of the regulation effects in the LPP amplitude. It is important to note that the observed pattern resembles some previous emotion regulation studies that have revealed numerically or significantly enhanced LPP amplitudes for down-regulation [27, 48, 57, 76] and numerically reduced LPP amplitudes for up-regulation [46] as well. There are several potential factors that could have caused these unexpected effects in the current and previous studies, such floor and ceiling effects [27, 46, 48], presentation of regulation instructions in a blocked rather than an intermixed fashion [47, 78], letting participants choose between different regulation strategies [57], or switching between up- and down-regulation. More research is needed to systematically test these and other factors to better understand the effects of regulation task characteristics on the LPP amplitude. For example, the first author is currently working on studies testing whether floor and ceiling effects cause the unexpected effects of emotion regulation instructions on the LPP. In addition, future studies could directly compare the effects of presentation of regulation instructions in a blocked versus an intermixed fashion, and the effects of letting participants choose between different regulation strategies versus instructing them to use one particular strategy. In addition, it would be informative to compare the effects of having participants perform both up- and down-regulation in a testing session versus only one of the two. Studies like those will provide more information about what exactly the LPP amplitude reflects in regulation tasks. Depending on the conclusions of those studies, the LPP amplitude may or may not have limited usability as a measure of regulation success. Alternative measures that could have merit as an objective measure of love regulation success in future studies are behavioral measures, skin conductance, heartbeat-related measures [79], facial electromyography [80], and activation of brain regions that have been associated with love [81].

To conclude, to the best of our knowledge, this is the first study concerning explicit regulation of love feelings. We argue that love regulation targets actual love feelings and we recognize that that in turn may affect emotions and relationship characteristics. The results showed that people have the preconception that love is somewhat uncontrollable. Nevertheless, they use various behavioral and cognitive strategies to cope with romantic break-ups and to maintain long-term relationships. In the context of heartbreak, distraction was used to feel better after a break-up (i.e., emotion regulation), while reappraisal was used to down-regulate love feelings. In the context of long-term relationships, communication/honesty was important for maintaining long-term relationships, while undertaking (new) activities with the beloved was used to prevent love feelings from declining (i.e., love up-regulation). These preconceptions of, and strategies for love regulation were replicated in two independent samples. Importantly, people were able to up-regulate their love feelings by thinking about the positive aspects of their partner and/or relationship and imagining positive future scenarios. People were also able to down-regulate their love feelings by thinking about negative aspects of their partner and/or relationship and imagining negative future scenarios.

This study, being the first of its kind, provides many suggestions for future research. In this study, we only tested the short-term effects of love regulation. For daily life applicability, it would of course be important that the effects of love regulation are long-lived and/or that people are able to perform love regulation habitually to obtain a sustained effect. Therefore, future studies should examine the long-term effects of love regulation, including its effects on well-being and relationship stability and satisfaction, as well as ways in which love regulation can become habitual. It would also be interesting to examine the effectiveness of behavioral and cognitive strategies other than reappraisal for regulating love feelings, including distraction, avoidance, and undertaking (new) activities with the beloved. In addition, it is important dissociate the effects of love regulation on love feelings and on affect, as a desirable effect on love feelings may be accompanied by an undesirable effect on affect, or vice versa. Love up- and down-regulation have numerous applications, ranging from stabilizing long-term relationships including marriages, reducing heartbreak after romantic break-ups, ameliorating unwanted crushes and forbidden loves, and perhaps even coping with the death of a beloved. In short, love regulation may increase the positive effects and decrease the negative effects of love on individuals and on society and therefore deserves much attention from the scientific community.

## Supporting Information

S1 Appendix Questions about perceived control over love feelings. (DOCX)

S1 Text Neutral IAPS pictures. (DOCX)
