# Supplementary material for: Regulation of Romantic Love Feelings: Preconceptions, Strategies, and Feasibility
Source: PLoS One. 2016 Aug 16;11(8):e0161087. doi: 10.1371/journal.pone.0161087 (PMC4987042; doi:10.1371/journal.pone.0161087)
Supplement: S1 Appendix — (DOCX) [file pone.0161087.s001.docx]

**S1 Questions about perceived control over love feelings**

Seventeen questions were constructed to measure perceived control over love in general (items 3, 11), and over infatuation (items 2, 4, 6, 7, 9, 10, 12, 14, 15, 16) and attachment (items 1, 5, 8, 13, 17) specifically. The questions were also constructed to measure perceived control over one’s own feelings (items 4, 5, 7, 8, 10, 14, 16) and the perceived control of people in general (items 1, 2, 3, 6, 9, 11, 12, 13, 15, 17). Finally, the questions probed perceived control over the intensity (items 1, 4, 5, 8, 10, 12) and the object (i.e., the specific person) of love feelings (items 2, 6, 7, 14, 16). Three questions contain a blank, in which participants mentally insert the name of their beloved. Six items (items 3, 9, 11, 13, 15, 17) are reverse coded.

|  |  | totally disagree |  |  |  | neutral |  |  |  | totally agree |
| --- | --- | --- | --- | --- | --- | --- | --- | --- | --- | --- |
| 1. | People can control how attached to someone they are. | 1 | 2 | 3 | 4 | 5 | 6 | 7 | 8 | 9 |
| 2. | People can control whether they fall in love with someone. | 1 | 2 | 3 | 4 | 5 | 6 | 7 | 8 | 9 |
| 3. | Love is uncontrollable. | 1 | 2 | 3 | 4 | 5 | 6 | 7 | 8 | 9 |
| 4. | I can control how infatuated with ___ I am. | 1 | 2 | 3 | 4 | 5 | 6 | 7 | 8 | 9 |
| 5. | I can control how attached to someone I am. | 1 | 2 | 3 | 4 | 5 | 6 | 7 | 8 | 9 |
| 6. | People can control who they fall in love with. | 1 | 2 | 3 | 4 | 5 | 6 | 7 | 8 | 9 |
| 7. | I can control whether I fall in love with someone. | 1 | 2 | 3 | 4 | 5 | 6 | 7 | 8 | 9 |
| 8. | I can control how attached to ___ I am. | 1 | 2 | 3 | 4 | 5 | 6 | 7 | 8 | 9 |
| 9. | Infatuation is uncontrollable. | 1 | 2 | 3 | 4 | 5 | 6 | 7 | 8 | 9 |
| 10. | I can control how infatuated with someone I am. | 1 | 2 | 3 | 4 | 5 | 6 | 7 | 8 | 9 |
| 11. | Love is involuntary. | 1 | 2 | 3 | 4 | 5 | 6 | 7 | 8 | 9 |
| 12. | People can control how infatuated with someone they are. | 1 | 2 | 3 | 4 | 5 | 6 | 7 | 8 | 9 |
| 13. | Attachment is uncontrollable. | 1 | 2 | 3 | 4 | 5 | 6 | 7 | 8 | 9 |
| 14. | I had control over whether I fell in love with ___. | 1 | 2 | 3 | 4 | 5 | 6 | 7 | 8 | 9 |
| 15. | Infatuation is involuntary. | 1 | 2 | 3 | 4 | 5 | 6 | 7 | 8 | 9 |
| 16. | I can control who I fall in love with. | 1 | 2 | 3 | 4 | 5 | 6 | 7 | 8 | 9 |
| 17. | Attachment is involuntary. | 1 | 2 | 3 | 4 | 5 | 6 | 7 | 8 | 9 |
