# Supplementary material for: Regulation of Romantic Love Feelings: Preconceptions, Strategies, and Feasibility
Source: PLoS One. 2016 Aug 16;11(8):e0161087. doi: 10.1371/journal.pone.0161087 (PMC4987042; doi:10.1371/journal.pone.0161087)
Supplement: S1 Text — (DOCX) [file pone.0161087.s002.docx]

**S1 Neutral IAPS pictures**

IAPS pictures used: 2037, 2038, 2102, 2191, 2235, 2305, 2358, 2383, 2393, 2396, 2397, 2435, 2487, 2506, 2512, 2518, 2560, 2575, 2579, 2580, 2593, 2594, 2595, 2635, 2745.1, 2749, 2850, 2890, 7550, 7620.
